# Mr. Loudhouter's Case of Hydrophobia

**Published:** 1808-10

**Authors:** W. H. Loudhouter

**Affiliations:** Flushing, Cornwall


### *To the Editors of the Medical and Physical Journal.*

Gentlemen,

THE following singular case of a paroxysm of Hydrophobia, which lately fell under my observation, I have submitted to you, requesting you will have the goodness to give it a place in your Journal.

I am, &c.

W. H. LOUDHOUTER.

*Flushing, Cornwall.*

---

### CASE.

On the 22d of July, 1808, Mr. H. a young man of about 25 years of age, made application to me on account of a violent liver complaint, which had been existing for above six months; previous to my seeing him, he had been under a course of mercury for above five weeks. From the appearance of symptoms at this time, I judged it expedient to continue the mercury, keeping him just on the verge of salivation, and at the same time ordering an opiate at bed time. This plan was continued until the 27th ult. when finding his mouth a little sore, he desisted from the mercurials until the 30th; the 31st he again commenced them. About the middle of this day he complained of a soreness of his throat; on examination I found the uvula and fauces inflamed in a very trifling degree. Supposing this to proceed from a slight cold, caught during the action of the mercury, I ordered him a very slight astringent gargle; during the course of the day, he several times complained of his throat, and that he found some difficulty in swallowing; notwithstanding this, he ate his dinners with apparently the same appetite he did when I first saw him, excepting a depression of spirits, (which I should have observed) had existed for two or three days.

In

In the afternoon he complained of a violent oppression of his chest, with an unpleasant titilating sensation in his throat; during the time of my examining his throat he fainted, and remained in a comotose state for about five minutes, when throwing some water forcibly in his face, he instantly recovered himself, and nearly sprung from the persons who were holding him, to catch the attendant whom he saw holding the glass, with the remains of the water in it; however, in this he was prevented; he was then attacked with violent convulsions from the lower part of the thorax upwards to the fauces, foaming at the mouth, with thick stertorous kind of breathing, universally attendant on hydrophobia. In this state he remained fifteen minutes, requiring three men to hold him; he then sunk down totally exhausted, remaining some time in this manner. I applied the usual stimulating applications to the temples, nostrils, &c. Finding these ineffectual, I determined again to have recourse to the cold water, in order to see whether a similar effect to the former would be produced; the consequences were the same, but in a more violent and instantaneous degree; this paroxysm continued about the same time as the first, he then went off in the same manner. The gentlemen around him, who were all strangers to him as well as myself, asked me my opinion of the case; to this, I with some diffidence answered, from the symptoms which we had been witnesses to, I had every reason to believe it to be a case of Hydrophobia.

I had scarce spoke the words, when he aroused from his lethargy, and with a vacant stare looked at every one round him; at length he broke out in the following exclamations, "Jones's young dog has bit me through my boot; it is nothing at all, do not be the least uneasy about it, I am not, but depend upon it I will shoot him the first opportunity; do not be offended." After several violent struggles, attended with a risus sardonicus, he fell into his former comatose state; in this interval, I prepared him an antispasmodic draught of opium, musk, &c. Just as he begun to recover himself, he burst into a violent flood of tears, which appeared to relieve him much, and bring him in a great measure to his recollection. I now thought it a favourable opportunity of offering him the draught, but in doing this the same effect as before was produced. My previous opinion of the case I now thought sufficiently formed, but I had to receive a still further confirmation. Just as he begun to recover himself, as before, he burst forth a second time, "Ponto, Ponto, my dog, why do

 you you not come to me? You used to be very fond of me, but now you will not come near me. Jones, the dog has bit me, but I do not care: I shall shoot him! He then threw himself on the pillow, and slept for twenty minutes; when he awoke, he appeared much more collected, but said he had been to sleep all the afternoon, and had had very unplesant dreams; but we could by no means get from him what they were. On asking him if he would take any thing, he replied, some coffee; this was brought; he appeared startled at the first sight of it, but afterwards took it with an avidity beyond conception; after this, he several times asked for beer instead of water. The first time I got him to take the antispasmodic draught; from this he slept about an hour; when he awoke, he said his throat was much better, he could swallow without difficulty, and complained of nothing but a slight pain in his head; he then took some water in a glass, which he swallowed without difficulty or dread; after this he enjoyed as good a night as he had done for some time, but now and then complained of his head being very hot; his pulse during the whole time were little more than natural; a blister was proposed but rejected; the extreme state of debility he had been reduced to by previous evacuations, and the mercury, forbid the use of the lancet. Antispasmodics were continued every three hours during the night.

August 1. Complains much of his side, but says his head and throat are perfectly well; during the whole day the patient has appeared in a very low melancholy state, but what little conversation he has held, has been perfectly rational. From this to the 31st inst. when he left me, no return whatever of any hydrophobic symptoms had appeared.

From the above statement of facts, I think little doubt can be entertained of this being a case of genuine hydrophobia.

Query.—Had the diseased action of the liver, or the affection of the sytem from mercury, any effect in counteracting the progress of the disease.

